# *Helicase-like transcription factor*-deletion from the tumor microenvironment in a cell line-derived xenograft model of colorectal cancer reprogrammed the human transcriptome-S-nitroso-proteome to promote inflammation and redirect metastasis

**DOI:** 10.1371/journal.pone.0251132

**Published:** 2021-05-19

**Authors:** Rebecca A. Helmer, Raul Martinez-Zaguilan, Gurvinder Kaur, Lisa A. Smith, Jannette M. Dufour, Beverly S. Chilton

**Affiliations:** 1 Department of Cell Biology & Biochemistry, Texas Tech University Health Sciences Center, Lubbock, Texas, United States of America; 2 Department of Cell Physiology & Molecular Biophysics, Texas Tech University Health Sciences Center, Lubbock, Texas, United States of America; 3 Department of Medical Education, Texas Tech University Health Sciences Center, Lubbock, Texas, United States of America; 4 Department of Pathology, Texas Tech University Health Sciences Center, Lubbock, Texas, United States of America; 5 Cancer Center, School of Medicine, Texas Tech University Health Sciences Center, Lubbock, Texas, United States of America; University of Hawai’i at Manoa, UNITED STATES

## Abstract

Methylation of the *HLTF* gene in colorectal cancer (CRC) cells occurs more frequently in men than women. Progressive epigenetic silencing of *HLTF* in tumor cells is accompanied by negligible expression in the tumor microenvironment (TME). Cell line-derived xenografts (CDX) were established in control (*Hltf*^+/+^) and *Hltf*-deleted male *Rag2*^*-/-*^*IL2rg*^*-/-*^ mice by direct orthotopic cell microinjection (OCMI) of *HLTF*^*+/+*^HCT116 Red-FLuc cells into the submucosa of the cecum. Combinatorial induction of *IL6* and *S100A8/A9* in the *Hltf*-deleted TME with *ICAM-1* and *IL8* in the primary tumor activated a positive feedback loop. The proinflammatory niche produced a major shift in CDX metastasis to peritoneal dissemination compared to controls. Inducible nitric oxide (*iNOS*) gene expression and transactivation of the *iNOS-S100A8/A9* signaling complex in *Hltf*-deleted TME reprogrammed the human S-nitroso-proteome. POTEE, TRIM52 and UN45B were S-nitrosylated on the conserved I/L-X-C-X_2_-D/E motif indicative of iNOS-S100A8/A9-mediated S-nitrosylation. 2D-DIGE and protein identification by MALDI-TOF/TOF mass spectrometry authenticated S-nitrosylation of 53 individual cysteines in half-site motifs (I/L-X-C or C-X-X-D/E) in CDX tumors. POTEE in CDX tumors is both a general S-nitrosylation target and an iNOS-S100A8/A9 site-specific (Cys^638^) target in the *Hltf*-deleted TME. *REL* is an example of convergence of transcriptomic-S-nitroso-proteomic signaling. The gene is transcriptionally activated in CDX tumors with an *Hltf*-deleted TME, and REL-SNO (Cys^143^) was found in primary CDX tumors and all metastatic sites. Primary CDX tumors from *Hltf*-deleted TME shared 60% of their S-nitroso-proteome with all metastatic sites. Forty percent of SNO-proteins from primary CDX tumors were variably expressed at metastatic sites. Global S-nitrosylation of proteins in pathways related to cytoskeleton and motility was strongly implicated in the metastatic dissemination of CDX tumors. *Hltf*-deletion from the TME played a major role in the pathogenesis of inflammation and linked protein S-nitrosylation in primary CDX tumors with spatiotemporal continuity in metastatic progression when the tumor cells expressed HLTF.

## Introduction

Metastasis is responsible for nearly 90% of deaths from cancer [1]. This estimate has been constant for more than 50 years, and CRC—the third most common cancer diagnosed in men and women worldwide [2]—is a prototypical example. Early screening tools, treatment modalities and lifestyle changes have improved survival. However, metastasis remains the main cause of CRC-related mortality mainly because 20–25% of CRCs are metastatic at initial diagnosis [3]. In the United States, the 5-year survival statistic for individuals with metastatic disease is 14%, compared to those with regional (71%) and localized (90%) CRC [4], and mortality rates are higher in men than women [5]. Understanding the mechanism responsible for CRC progression and metastasis is essential to the design of treatments to improve patient prognosis.

HLTF protein—the multidomain mammalian ortholog of yeast Rad5—has a DNA-binding domain, a HIRAN domain, and a C3HC4-type RING-finger embedded between SNF2 helicase motifs [6]. HLTF cDNA was cloned based on the protein DNA-binding capabilities. The cloning was independently done by three research groups, Sheridan et al. [7], Hayward-Lester et al. [8] and Ding et al. [9] who initiated the use of the HLTF name. HLTF mediates replication fork reversal to rescue a stalled replication fork under stress via its HIRAN domain [10], and is a ubiquitin ligase targeting PCNA for polyubiquitination via its RING domain [11]. HLTF is a putative tumor suppressor because of its role in DNA damage tolerance mechanisms [12]. Epigenetic silencing of *HLTF* has been implicated in the malignant transformation of adenomas to carcinomas [13] because the *HLTF* gene is silenced by hypermethylation in 43% of colon cancers [14]. Methylated *HLTF* DNA in serum is significantly correlated with tumor size, more aggressive tumors, advanced stage (III or IV) metastatic disease, including micro-metastasis, and shorter survival [15–18]. Progressive loss of *HLTF* expression from tumor cells correlates with tumor staging [19]. There are higher levels of HLTF protein in tumors from patients with early-stage 1 CRC compared to patients with late-stage IV disease. In contrast, immunohistochemistry showed negligible HLTF protein in fibroblasts of the tumor microenvironment (TME) regardless of tumor stage. These findings were reinforced by reference component RNAseq analysis of single-cell transcriptomes from eleven primary tumors and matched normal mucosa [20]. Three fibroblast populations—normal, myofibroblasts, and cancer-associated fibroblasts—were identified based on gene expression profiles. *HLTF* was not detected in any of the fibroblast populations despite measured changes in mRNA levels between normal and tumor cells. Epigenetic silencing of *HLTF* in tumor cells has long been the experimental focus to the exclusion of *HLTF* in the TME [21].

CRC progression is accompanied by overexpression of nitric oxide (NO)—a diffusible/pleiotropic regulator of many normal cellular processes—implicated in the activation of oncogenic signaling pathways [22, 23]. NO is endogenously generated by three different isoforms of the enzyme NO synthase (NOS) using L-arginine and molecular oxygen as substrates. Of the three nitric oxide synthase (NOS) isoforms, neuronal (nNOS, NOS1) and endothelial (eNOS, NOS3) are calcium-dependent isoforms that produce nanomolar concentrations of NO for seconds or minutes when activated. In contrast, the inducible (iNOS, NOS2) isoform is calcium-independent. and produces micromolar concentrations of NO for hours or days [24]. NO induces post-translational coupling of a nitroso moiety to a reactive cysteine leading to protein S-nitrosylation that affects protein-protein interactions [25]. This pathway is independent of the NO/soluble guanylate cyclase (sGC)/cGMP-dependent protein kinase (PKG) signaling pathway. The NO signal can be stored and propagated at nitrosyl adducts at specific cysteine sites of proteins. S-nitrosylation is purported to be site-selective. INOS-S100A8/A9 targets a conserved I/L-X-C-X_2_-D/E motif [26]. However, S-nitrosylation is a reversible covalent chemical reaction and therefore one of the more difficult post-translational modifications to study. Progress in this regard is owed to the three fundamental steps of the biotin-switch assay [27]: free sulfhydryls are chemically blocked, nitrosylated cysteines are selectively reduced, and a biotin adduct is switched for the NO adduct. Recent modifications substitute iodoTMT reagents [28] in the biotin-switch procedure and use mass spectrometry to identify and quantify specific S-nitrosylated residues.

To investigate the functional importance of negligible HLTF expression in fibroblasts of the TME, we developed an HCT116 cell line-derived xenograft model of metastatic CRC. Primary tumor xenografts were established in *Hltf*-deleted and control male mice by direct OCMI of *HLTF*^*+/+*^HCT116 Red-FLuc cells [29]. The HCT116 human colon carcinoma cell line was selected because it expresses HLTF [30], displays leading edge invasion in xenograft tumor model [31], and recapitulates the multi-step dissemination process to the liver and lungs [32]. However, *HLTF*^*+/+*^HCT116 cells in *Hltf*-deleted mice shifted their metastatic direction. Species-specific RNAseq analysis of primary CDX tumors arising from the same passage of *HLTF*^*+/+*^HCT116 Red-FLuc cells in the TME of *Hltf*-deleted and control mice revealed striking coordination of an inflammation pathway. Transactivation of iNOS expression and the iNOS-S100A8/A9 signaling axis in *Hltf*-deleted TME drove the comparison of S-nitroso-proteome of primary tumor with metastatic tumors in *Hltf*-deleted TMEs to reveal remarkable spatiotemporal continuity in S-nitrosylation signaling.

## Results

### Experimental timeline ensured mouse survival

**Fig 1:**
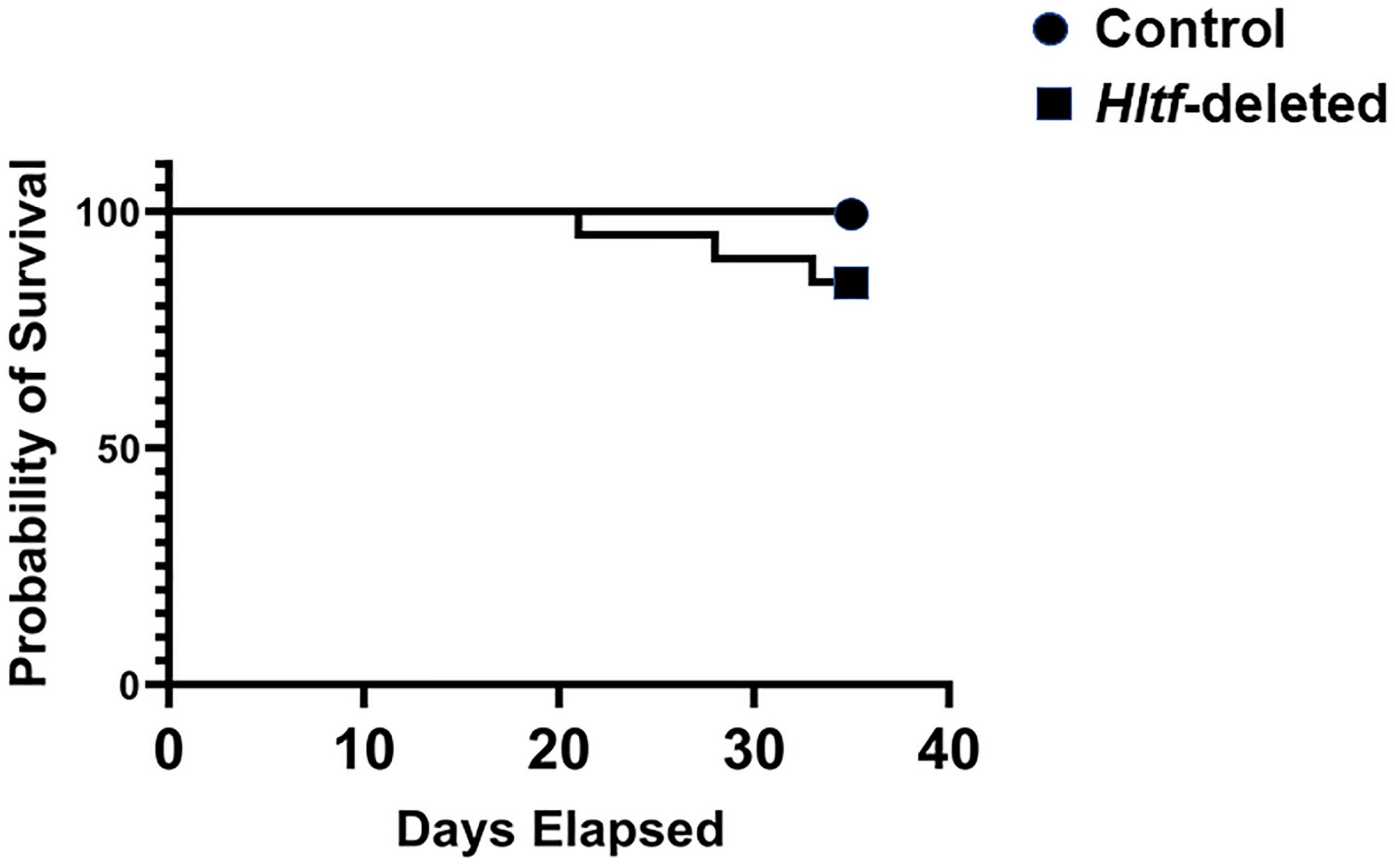
Kaplan-Meier survival plot. Comparison of the cumulative survival curve of male *Hltf-*deleted (n = 20) and control (n = 12) mice shows the total probability of the mice surviving to the end of the study, i.e. 35 days post-surgery, was unaffected by genotype.

There is a negative correlation between HLTF expression in tumor cells and survival in mice [33]. Therefore, when *HLTF*^*+/+*^ human HCT116 Red-FLuc cells were used to establish an orthotopic xenograft model in *Hltf*^*+/+*^ (control mice) and *Hltf*-deleted mice a timeline of 35 days was established during which primary tumor size and metastasis was assessed weekly by BLI. At necropsy, primary tumors were ≤ 10 mm, i.e. below the maximum allowable tumor size of 20 mm tumor development/metastasis did not interfere with daily activities (eating, drinking, nest-building, locomotor). Additionally, none of the mice experienced bowel obstruction or changes in stooling or stool consistency. There was no evidence of blood associated with stooling. There was no evidence of anemia, i.e. loss of pink condition of mouse footpads. There was no evidence of compromised behavior, i.e. mice did not become lethargic and there was no incidence of piloerection, poor grooming or inability to thermoregulate. The mice experienced no sustained weight loss. Comparison survival curves for *Hltf-*deleted mice (n = 20) and control mice (n = 12) mice with the logrank (Mantel-Cox) test (Chi square 1.898, p = 0.1683), and the Gehan-Breslow-Wilcoxon test (Chi square 1.895, p = 0.1687), indicated *Hltf*-deletion had no significant effect on the mortality of the mice during the 35-day post-surgery timeline (Fig 1). However, three *Hltf*-deleted mice died. Thus, the hazard ratio (Mantel-Haenszel) indicated *Hltf*-deleted mice at any time during the treatment protocol were 5-times more likely to die than control mice. This approach determined an earlier humane endpoint of 28 days for *Hltf*-deleted mice in future experiments. The mice were treated alike and maintained under identical conditions.

### HLTF expression is negatively associated with survival in human CRC

**Fig 2:**
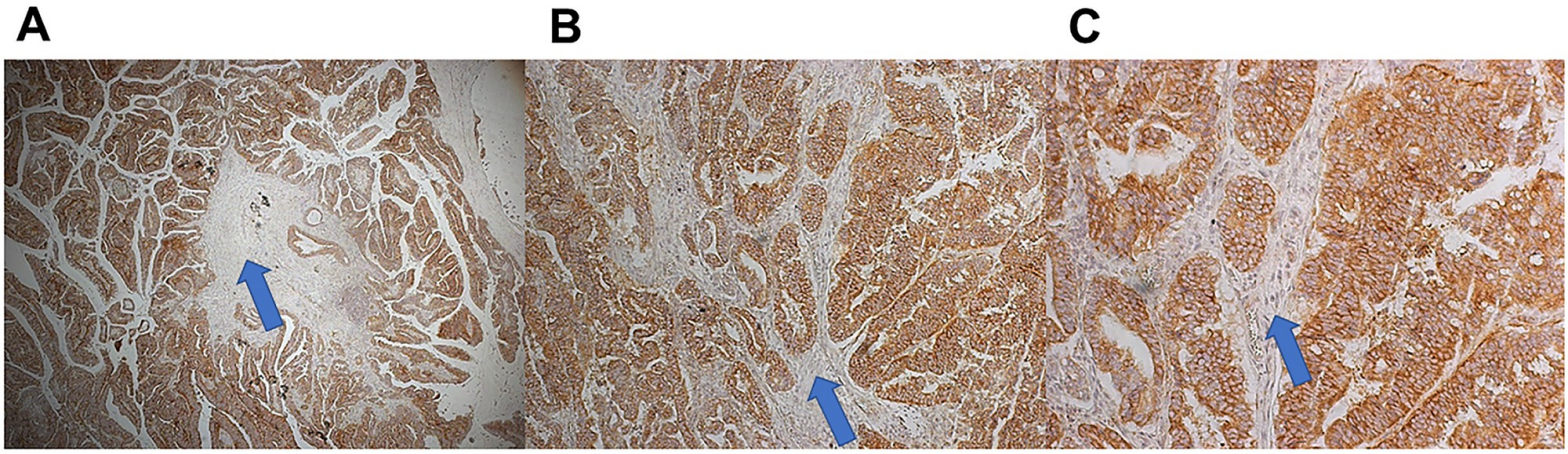
HLTF protein in a pT3 adenocarcinoma with no regional lymph node metastasis (pN0). HLTF-immunostaining in well-differentiated (G1) cancer cells of an adenocarcinoma that arose from a tubulovillous adenoma (polyp). Tumor cells invaded through the muscularis propria into the peri-colorectal tissue at the hepatic flexure. There was no evidence of lymphovascular invasion. The photomicrographs in panels A-C show the cellular architecture of the tumor tissue composed of HLTF-positive cancer cells and HLTF-negative fibroblasts. A (4X magnification), B (10X magnification) and C (20X magnification). Antibodies to the HLTF N-terminus (residues 164–300) that are common to all known human HLTF proteins [21] in nuclear and cytoplasmic locations show the positive immunostain (brown) is predominately although not exclusively cytoplasmic. HLTF-negative fibroblasts (blue arrows) are devoid of nuclear and/or cytoplasmic staining.

There is a negative correlation between HLTF expression and the progression of CRC in humans [11–16]. Previous studies have shown the progressive loss of HLTF from tumor cells correlates with negligible HLTF expression in the TME [19, 20]. The CDX model is predicated on the paucity of HLTF expression in the fibroblasts of the TME as shown in a well-differentiated adenocarcinoma (Fig 2) from a male patient that resulted in hemicolectomy.

### *Hltf-*deletion from the TME promotes metastasis in the CDX model

**Fig 3:**
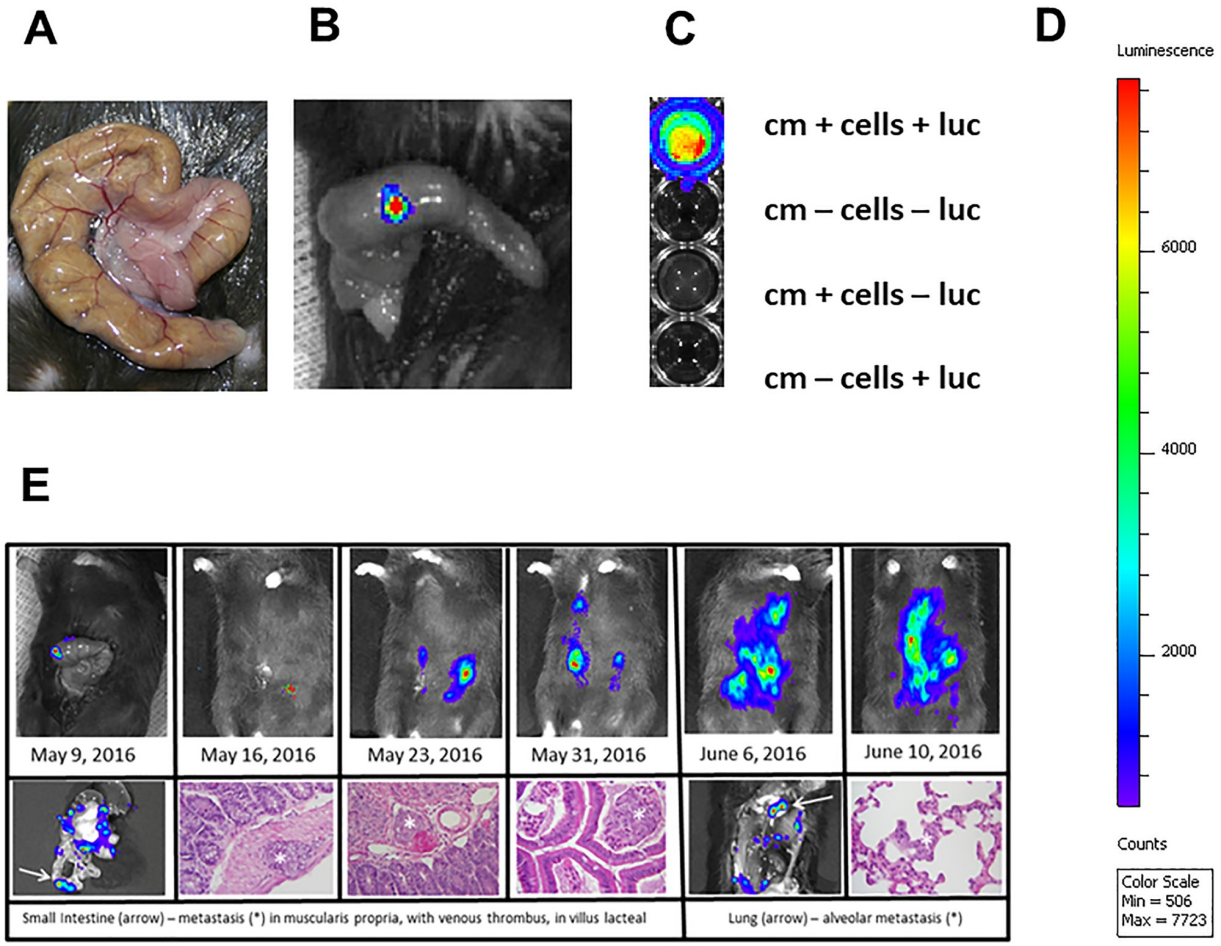
Direct orthotopic cell microinjections (OCMI). Control mice received direct OCMI of 2x10^6^ *Hltf*^+/+^ HCT116 Red-FLuc cells/10 μl into the submucosa of the variable size (3–4 cm) J-shaped cecum (A). *In vivo* BLI (one-second exposure) followed IP injection of D-luciferin (B). A 50 μl Hamilton syringe with 30GA/0.5 inch/30-degree custom needles eliminated retrograde leakage. Cell signal authenticity (C) was confirmed *in vitro* (cm = culture medium, luc = luciferin). BLI intensity (D) ranged from red (highest), through yellow, green, blue, and purple (lowest). Histopathological confirmation with Hematoxylin and Eosin staining of tumor foci in the most clinically relevant metastatic sites coincides with BLI (E) in control mice. BLI and histopathology showing progression in the same mouse.

**Fig 4:**
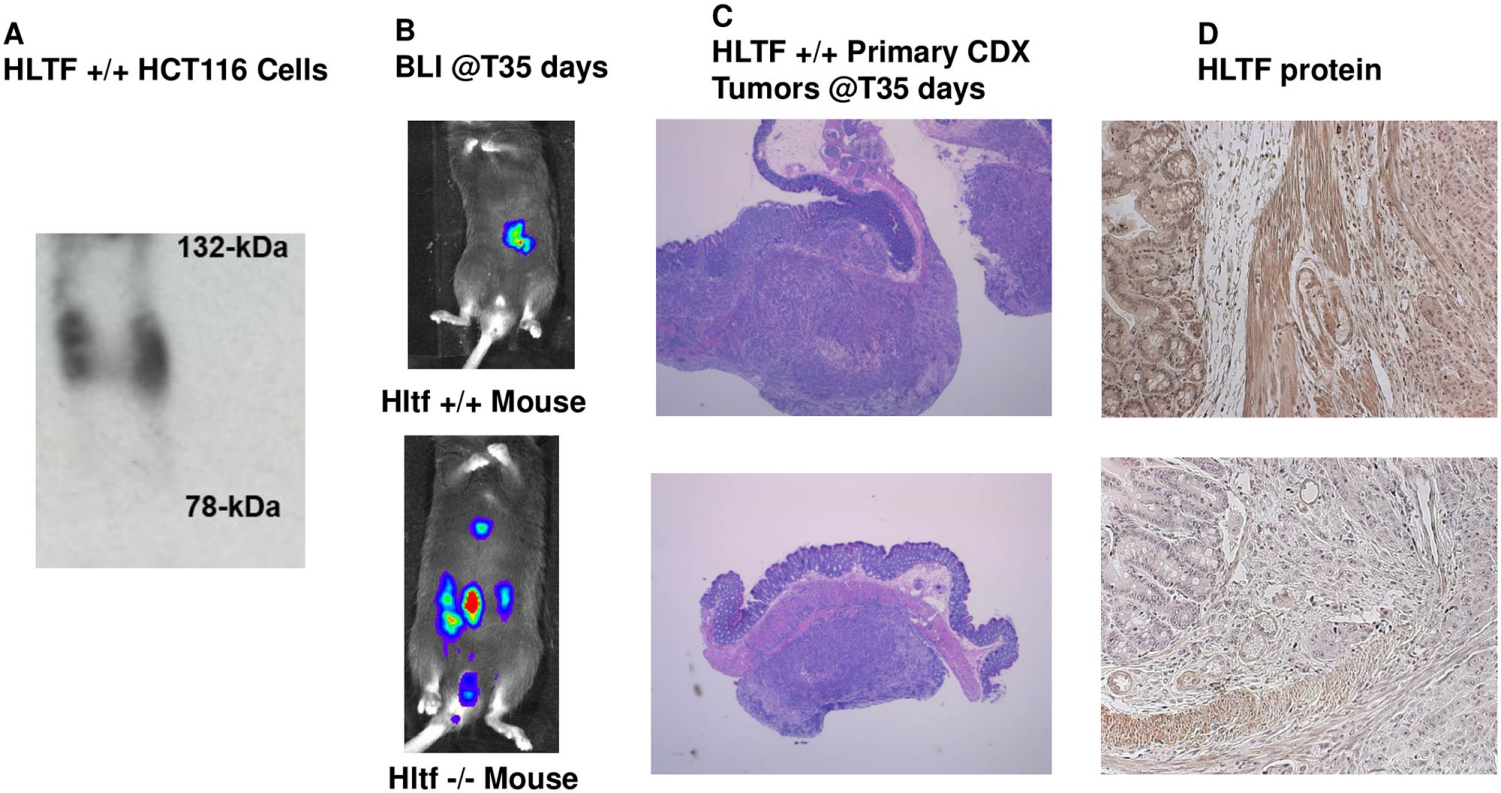
Representative images of immunodetection, BLI, routine histochemistry and HLTF-immunohistochemistry. Full-size HLTF protein (~115-kDa) was detected by Western blot of immunoprecipitated proteins from HCT116 Red-FLuc cells (A). The HLTF antibodies (residues 600–700) recognize all known and putative truncated mouse proteins [35]. BLI showed the metastatic properties (local invasion and distant colony formation) of primary tumor xenografts dramatically increased in an *Hltf*-deleted microenvironment (A). At necropsy, primary xenograft CDX tumors (B) were removed for routine histopathology (Hematoxylin and Eosin staining; 4X magnification). Primary xenograft CDX tumors (C) showed positive immunostaining for HLTF. Immunostaining in the *Hltf*^*+/+*^ TME contrasts with the *Hltf*-deleted TME (25 X magnification). The HLTF antibodies (residues 164–300) recognize all known human and mouse proteins [35].

**Fig 5:**
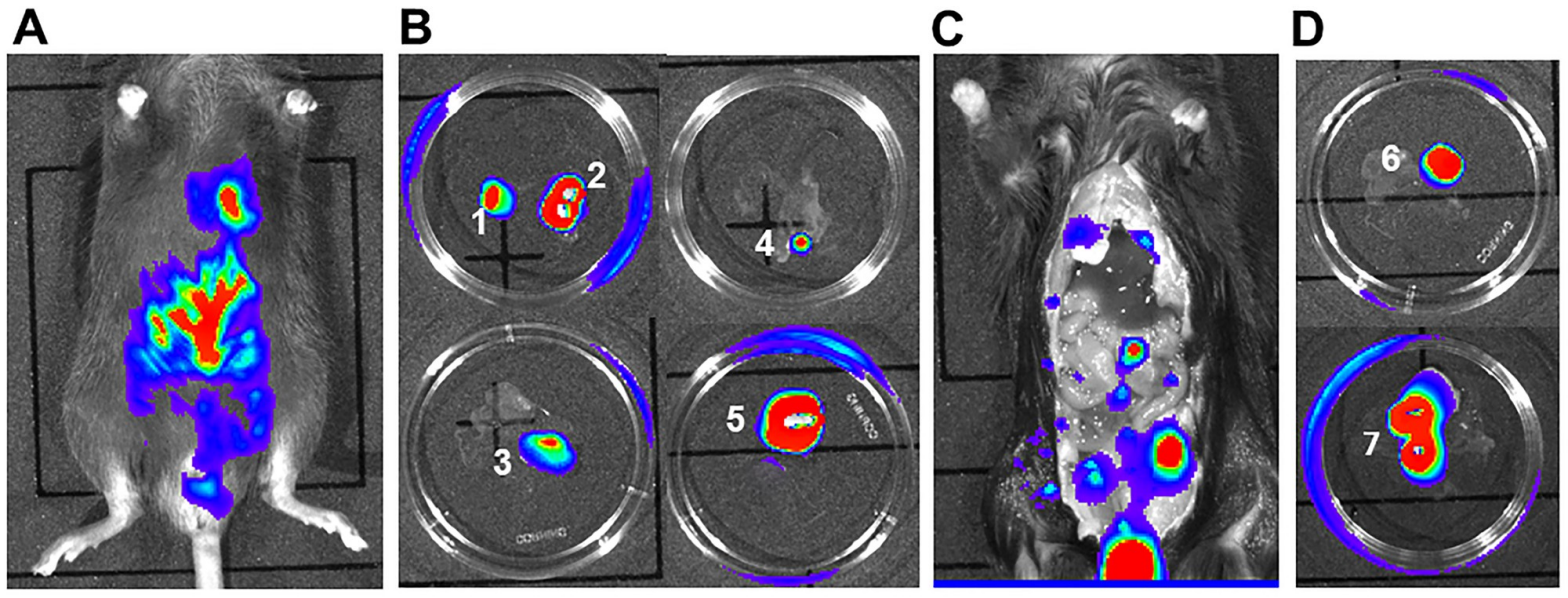
Imaging of CDX metastasis in *Hltf-*deleted mice at necropsy. Mice were anesthetized with isoflurane and metastatic CRC was detected by BLI (A) prior to necropsy. Immediately following euthanasia, mice were opened via a mid-sagittal incision and the primary CDX tumor (B1) was removed followed by lymph nodes near the stomach and spleen (B2). Metastatic sternal lymph nodes below (B3) and above (B4) the diaphragm were removed as was a peritoneal carcinomatosis (B5). Mice were imaged a second time (C) to guide removal of inguinal lymph nodes (D6) and metastatic tissue extending into the scrotum (D7). All tissues were evaluated for S-nitrosylation of human proteins.

The goal of the first experiment was to establish primary tumor xenografts with HLTF expressing HCT116 Red-FLuc cells (easily detected by bioluminescence imaging—BLI) in control male mice by direct OCMI between the mucosa and the muscularis layers of the cecal wall (Fig 3). Care was taken to avoid microinjection into the lymphoid nodules in the distal part of the mouse cecum. Histopathological confirmation of tumor foci in the most clinically relevant [32] metastatic sites (e.g., colon-draining lymphatics, liver, lung and peritoneum) coincided with BLI. The goal of the second experiment was to establish primary tumor xenografts via OCMI in Hltf-deleted mice. Tumor take rates were 100% for primary xenograft tumors for *Hltf*-deleted (n-20) and control (n = 12) mice. However, a shift in the pattern of metastases (Fig 4) to regional lymph nodes, peritoneal carcinomatosis (PC), inguinal canal in close proximity to seminal vesicles and into the scrotum occurred in *Hltf*-deleted TME (Fig 5). PC is a late stage manifestation of CRC and considered lethal in humans [34].

### The dynamic interaction between cells of the tumor and the TME was decoded by species-specific comparative transcriptomics

Alternative splicing increases the complexity of HLTF gene expression in human and mouse cells. Because of its putative role in tumor development, alternative splicing in cells of the tumor and TME were evaluated. Two transcript variants encode the same HLTF protein in *Homo sapiens* [21]. Cuff.diff alternative splicing analysis identified transcript variant 1 (NM_003071.4), the longer transcript variant (5320-bp mRNA), as the only protein (Fig 4A) encoding mRNA transcript in primary CDX tumor cells. Hltf is alternatively spliced in mouse tissues [33, 35–37], and Cuff.diff alternative splicing analysis quantified the usage of each exon and each possible splice junction for *Hltf* in RNA-seq samples from control mouse TME. A 4:1 ratio of full-length 4956 nucleotide (nt) message isoform (NM_09210) to a long non-coding (Lnc) 3643 nt transcript variant 4 (Lnc-Hltf-4; NR_105047) was identified. This implicates Lnc-Hltf-4 in CRC progression.

Cuff.diff FPKM tracking files were analyzed with iPathwayGuide (Advaita Bioinformatics)–using the q value of 0.05 for statistical significance and a log fold change of expression with an absolute value of at least 0.06–30 differentially expressed (DE) genes were identified out of a total of 15,857 genes with measured expression in the mouse TME (S1 Table). Similarly, for human tumor analysis–using the q value of 0.05 for statistical significance and a log fold change of expression with an absolute value of at least 0.06–151 DE genes were identified out of a total of 12,358 genes with measured expression in the human tumor (S2 Table).

**Fig 6:**
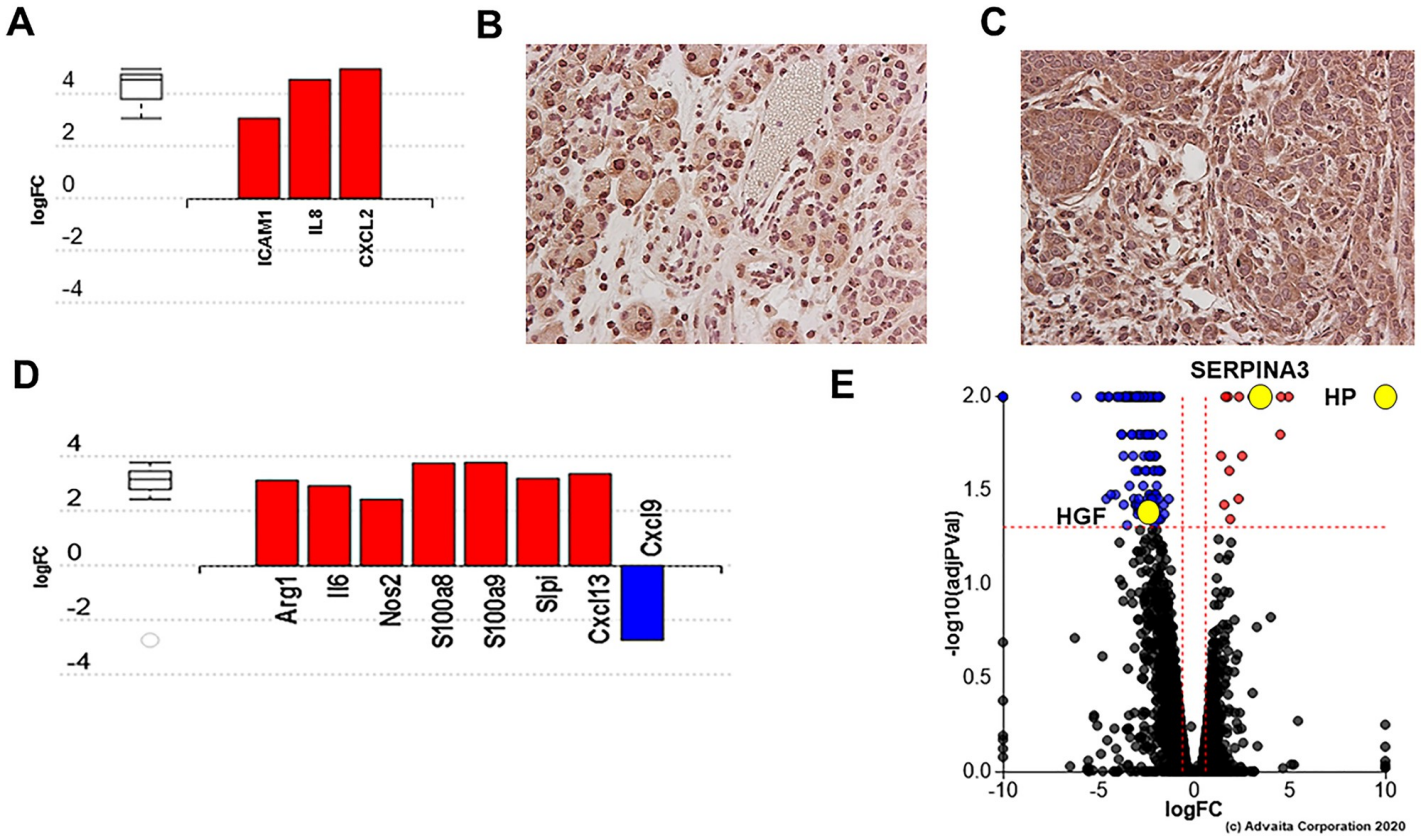
Gene measured expression bar plots and confirmatory immunohistochemistry. All DE genes are ranked based on their absolute log fold change. Upregulated genes are shown in red and down regulated genes are shown in blue. Box and whisker plots on the left of each histogram summarize the distribution of all DE genes in a specific pathway or annotated to a GO term out of all of the target genes. The box represents the first quartile, the median and the third quartile. Increased mRNA (A) and protein expression of pro-inflammatory cytokines, *IL8* (B) and *ICAM-1* (C), occurs in human tumors with an *Hltf*-deleted TME (40X magnification). There is no IL8 staining in the TME because *IL8* is unique to the human genome, i.e. there is no *IL8* homologue in the mouse. The log fold increase in message for *IL6* and all members of the iNOS-S100A8/A9 signaling axis was documented in the mouse *Hltf*-deleted TME (D). Log fold changes in message for *HP* (increase, p = 0.01), *SERPINA3* (increase, p = 0.01) and *HGF* (decrease, p = 0.043) in CDX tumors from *Hltf*-deleted TME are highlighted with yellow dots in a volcano plot (E). The horizontal axis is the log fold change, and the vertical axis is the negative base-10 logarithm of the p-value. The red-dotted lines represent the threshold. The upper regulated genes (positive long fold change) are shown in red, while the down-regulated genes are blue. Unaffected genes are black.

**Fig 7:**
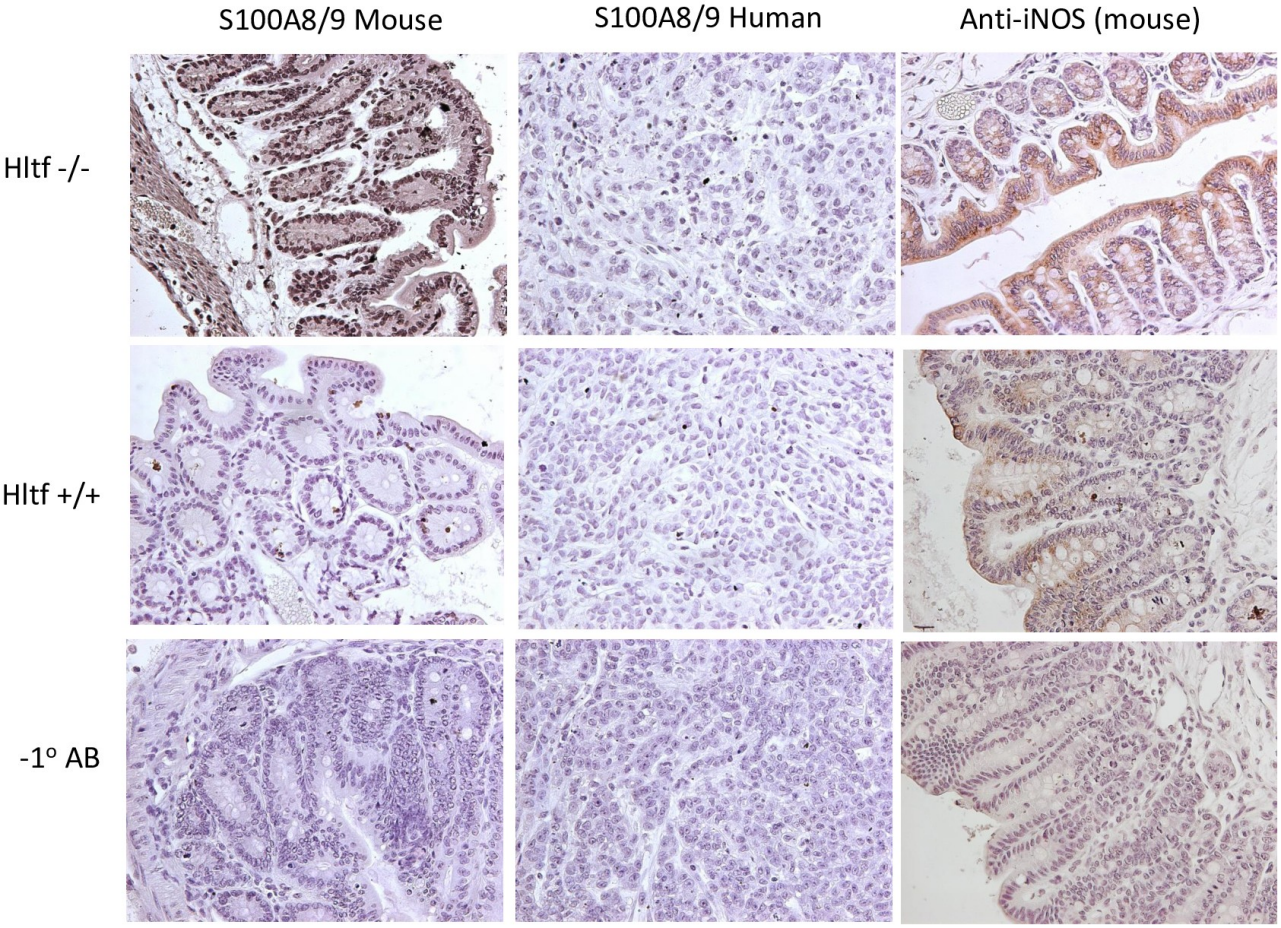
Immunohistochemistry for the iNOS-S100A8/A9 signaling axis. Increased S100A8/A9 was exclusive to the *Hltf*-deleted mouse TME. S100A8 and S100A9 typically form heterodimers (S100A8/A9, calprotectin) and homodimers are not generally detectable. Increased S100A8/A9 expression coincides with increased iNOS protein levels in the *Hltf*-deleted mouse TME. Increased endogenous iNOS expression, a major mediator of inflammation, correlates with poor patient survival in CRC due to increased metastasis.

Hypoxia is an important regulator in CRC, and Hltf is a transcriptional regulator of murine hypoxia inducible factor-1α (Hif-1α) in heart [36]. However, transcriptional availability of HIF1α in the CDX tumors was unaffected by the presence or absence of Hltf in the TME. In addition, of the 151 DE genes, only 14 of ~2450 TRANSFAC predicted HIF1α targets were either up (n = 3) or down (n = 11) regulated, and no functional links between them were identified by iPathwayGuide. These findings argue *Hltf*-deletion from the TME had little or no effect on the oxygenated/metabolic state of CDX tumors. In fact, cytokine-receptor interaction (KEGG: 04060) was the top biological pathway in CDX tumors (p = 0.002) and mouse TME (p = 2.546e-5) with Bonferroni corrected p-values. Increased mRNA (Fig 6A) and protein (Fig 6B and 6C) expression of the pro-inflammatory cytokines, interleukin 8 (*IL8*) and intercellular adhesion molecule-1 (*ICAM-1*) was identified in CDX tumors with an *Hltf-*deleted TME. Transactivation of interleukin 6 (*IL6*) and all components of iNOS-S100A8/A9 signaling in the *Hltf*-deleted TME (Fig 6D) coincided with dramatic increases in the mRNA abundance of haptoglobin (*HP*) and *SERPINA3*, and decreased hepatocyte growth factor (*HGF*) in CDX tumors (Fig 6E). Compartmentalized expression of all components of the iNOS-S100A8/A9 signaling pathway in the *Hltf*-deleted TME are shown in Fig 7.

### iNOS-S100A8/A9 targets in CDX tumors

We tested the hypothesis that iNOS derived nitric oxide (NO) production in the *Hltf*-deleted TME promoted metastasis in the CDX model via S-nitrosylation. A snapshot of the entire CDX tumor S-nitroso-proteome in the presence/absence of Hltf in the TME was obtained when iodoTMT enriched S-nitrosylated human proteins were interrogated by nanoLC-MS/MS. NanoLC-MS/MS-based protein identification provided a comprehensive collection of 136 SNO-proteins in CDX tumors with an *Hltf*-deleted TME compared to 178 SNO-proteins in CDX tumors with control TME (S1 File). Protein-protein interaction analysis with PANTHER designated cytoskeletal regulation by the Rho GTPase (P00016) the major pathway for CDX tumors from both *Hltf*-deleted and control TME. However, a secondary pathway of inflammation-mediated by chemokine and cytokine signaling (P00031) increased from 11 protein candidates to 18 protein candidates in CDX tumors with the *Hltf*-deleted TME. The emPAI value (0.28) for POTEE, the primate-specific POTE (**p**rostate, **o**vary, **t**estis and **e**mbryo expressed) ankyrin domain family member E that promotes CRC growth [38], indicates this protein is a general target of S-nitrosylation in CDX tumors from *Hltf*-deleted and control TME.

**Fig 8:**
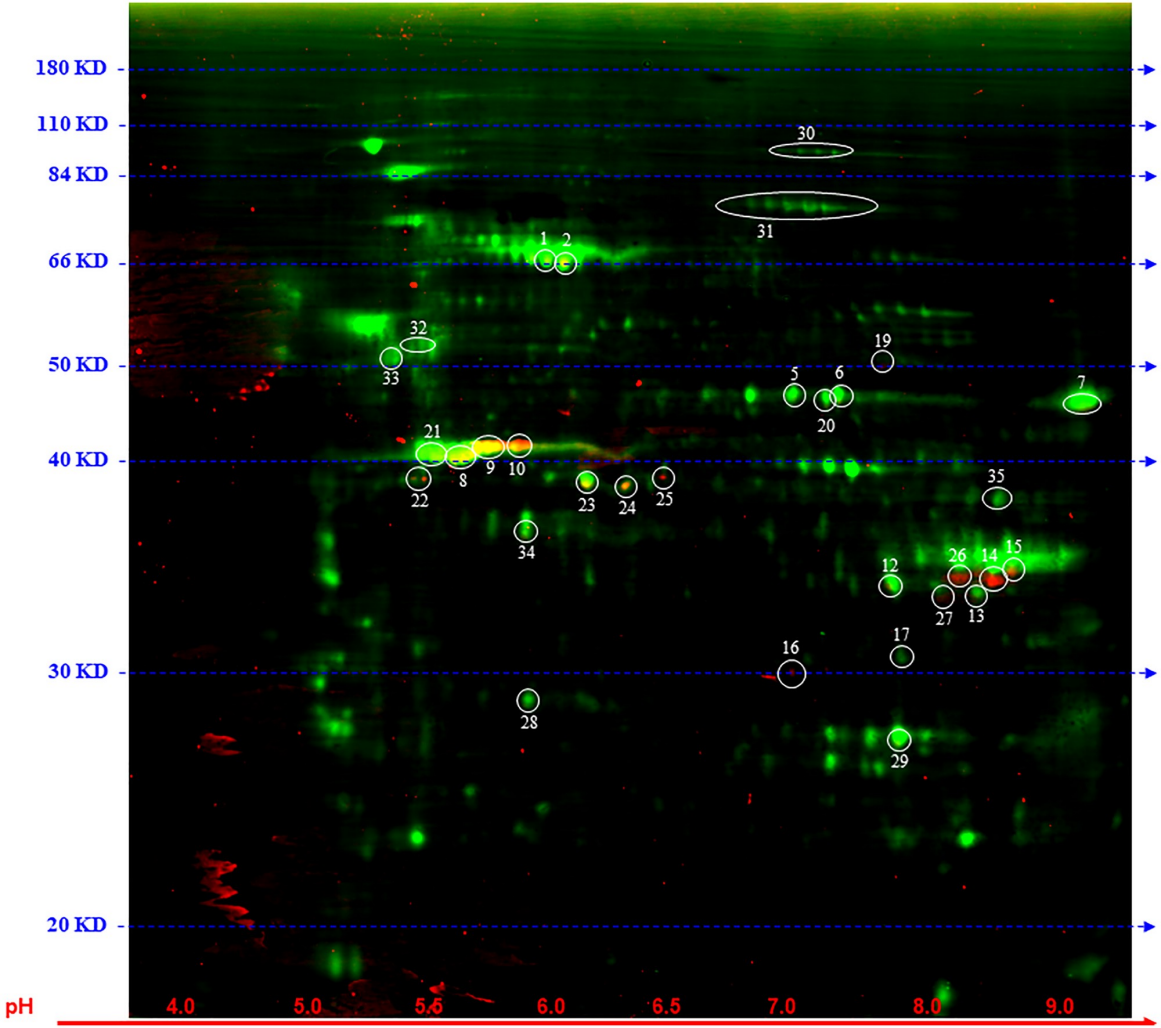
2D-DIGE gel electrophoresis. For purposes of data presentation, only select spots are indicated in the gel. The pH range 4 to 9 is indicated on the x-axis (red horizontal line). Molecular mass (20–180 KD) is indicated on the y-axis. Differentially expressed proteins were subjected to MALDI-TOF/TOF analysis.

Differential expression of TMT-labeled proteins was studied by 2DE with Cy5 label followed by 2D-DIGE (S2 File) and MALDI-TOF/TOF (S3 File). IodoTMT-labeled proteins from *Hltf*-deleted and control mice were covalently tagged with either Cy3 (*Hltf-*deleted) or Cy2 (control) for 2D-DIGE (Fig 8). A gel with three fluorescent images (Cy5/Cy2/Cy3) was processed for spot-codetection and protein identification by MALDI-TOF/TOF with a standard database search for human proteins with MASCOT. Proteins-peptides with a total ion C.I.% = 100 are presented in the protein ID summary.

2D-DIGE and protein identification by MALDI-TOF/TOF led to the authentication of iNOS-S100A8/A9 consensus site-specific S-nitrosylated proteins in CDX tumors from *Hltf*-deleted TME. As shown in Fig 8, spot 21 that is unique to CDX from *Hltf-*deleted TME has authentic S-nitrosylation of Cys^638^ in the consensus sequence motif (I/L-X-C-X-X-D/E) of three members (E/F/I) of the primate-specific POTE gene family comprised of 14 members and subdivided into four groups. Group 3 POTE-actin genes (POTEs **E/F/I**/J/KP) encode proteins that are actin chimeras with a full-sized long inverted repeat, seven ankyrin repeats and a C-terminal coiled-coil domain. The consensus iNOS-S100A8/A9 target sequence (aa 636–641) immediately precedes the start of the coiled-coil domain (aa 642–698). POTEE-SNO in CDX tumors is both a general S-nitrosylation target and an iNOS-S100A8/A9 site-specific target in the *Hltf*-deleted TME. When additional very weak spots (30–35) were added to the analysis, authentic S-nitrosylation of Cys^238^ in the consensus sequence motif (I/L-X-C-X-X-D/E) was identified in the tripartite motif (TRIM) family member TRIM52. Dysregulation of TRIM family members characterized by a tripartite motif–RING domain, one or two B-box domains, and a coiled-coil domain–has been implicated in CRC cell proliferation [39]. This newly identified iNOS-S100A8/A9 target sequence (aa 236–241) is located in the B-box domain. In contrast, all members of the ERM family—ezrin, radixin, and moesin—were found in spot 31 as general targets of S-nitrosylation (Cys^284^). There was no evidence of iNOS-S100A8/A9 site-specific S-nitrosylation (Cys^117^). 2D-DIGE and protein identification by MALDI-TOF/TOF led to the authentication of S-nitrosylation of 53 individual cysteines in half-site motifs of either I/L-X-C or C-X-X-D/E in proteins from CDX tumors from both *Hltf*-deleted and control TME (Table 1).

**Table 1:** S-nitrosylated cysteine residues (yellow) in half-site motifs of either I/L-X-C or C-X-X-D/E from CDX tumors from *Hltf*-deleted (white) and control (green) TME.

-2	-1	C	+1	+2	+3	Position	Spot #
I/L	X	**C**	X	X	D/E	Consensus	
L	T	C	R	L	E	GAPDH, Cys^247^	
L	S	C	K	K	E	POTE E/F/I, Cys^638^	21
L	F	C	E	V	D	TRIM52, Cys^238^	35
L	A	C	G	I	I	KCAB1, Cys^300^	3
I	Q	C	I	Q	A	TRFL, Cys^64^	3
L	L	C	E	L	L	LIMC1, Cys^55^	4
L	N	C	N	D	C	LIMC1, Cys^1067^	4
L	S	C	K	Q	L	PRKDC, Cys^1507^	4
L	D	C	D	L	K	ROCK2, Cys^766^	4
I	Y	C	R	S`	R	MED14, Cys^928^	6
I	S	C	T	L	N	BNC1, Cys^37^	6
L	N	C	S	C	Q	BNC1, Cys^41^	6
L	N	C	P	D	A	BNC1, Cys^307^	6
I	T	C	H	L	C	BNC1, Cys^930^	6
I	P	C	S	G	S	PPE2, Cys^377^	16
I	T	C	F	D	I	KI26B, Cys^2100^	16
L	L	C	D	M	T	RPB1, Cys^1402^	18
I	A	C	L	A	F	EMAL5, Cys^111^	18
L	L	C	s	F	R	DGKQ, Cys^607^	18
L	N	C	S	V	N	ITA6, Cys^967^	19
I	S	C	P	I	C	MYCBP2, Cys^4437^	26
I	Y	C	N	V	R	MYCBP2, Cys^114^	26
L	W	C	Y	N	A	MYCBP2, Cys^1111^	26
I	L	C	C	N	S	RFC3, Cys^165^	26
I	L	C	C	N	S	RFC3, Cys^164^	26
L	P	C	I	L	N	LDHB, Cys^294^	27
I	V	C	K	P	V	MRPL15, Cys^188^	27
I	I	C	D	V	C	ITB4, Cys^452^	27
L	K	C	V	G	H	ATS15, Cys^920^	29
I	Q	C	A	E	K	ECHM, Cys^225^	29
L	N	C	S	T	K	GLYL3, Cys^6^	30
I	S	C	F	P	S	GLYL3, Cys^187^	30
I	P	C	H	P	S	HOIL1, Cys^502^	30
L	C	C	L	R	Y	PIEZ1, Cys^479^	35
I	V	C	K	M	L	PIEZ1, Cys^868^	35
L	A	C	P	M	P	DYH11, Cys^4455^	35
F	K	C	L	R	D	TRFL, Cys^217^	3
A	L	C	I	D	D	TRFL, Cys^526^	3
S	R	C	I	E	D	TNG6, Cys^453^	4
V	E	C	W	K	D	PRKDC, Cys^2244^	4
H	K	C	T	I	E	BCN1, Cys^387^	6
K	A	C	G	S	E	DESP, Cys^1280^	11
D	L	C	R	L	D	MFR1L, Cys^52^	16
N	P	C	P	M	D	FBLN7, Cys^333^	18
R	P	C	D	S	E	EMAL5, Cys^1279^	18
A	H	C	L	Q	D	DYST, Cys^2476^	19
A	I	C	H	P	D	DYST, Cys^7993^	19
K	S	C	E	N	D	TRPC7, Cys^740^	20
M	R	C	T	T	D	MED16, Cys^267^	22
G	A	C	P	T	E	MED16, Cys^790^	22
-	M	C	D	E	D	ACTA1, Cys^2^	23
-	M	C	D	D	E	ACTC1, Cys^2^	24
D	D	C	I	C	D	PPEF2, Cys^696^	30
S	V	C	Y	R	D	GLYL3, Cys^192^	30
C	D	C	N	L	E	LAMA3, Cys^493^	35
Q	G	C	Q	C	D	LAMA3, Cys^684^	35

### Continuity between primary CDX tumors and metastatic sites

Comparative assessment of S-nitrosylation in primary CDX tumors and five metastatic sites was undertaken with iodoTMT-switch labeling and nanoLC-MS/MS. As shown in Fig 5, BLI was used to carefully isolate tumors and lymph nodes, and proteins were selectively labeled with iodoTMT isobaric label reagents as follows: iodoTMT-126, lymph nodes near stomach and spleen; iodoTMT-127, primary tumors; iodoTMT-128, peritoneal carcinomatosis; iodoTMT-129, tumors at the sternum (below and above the diaphragm); iodo-TMT130 lymph nodes in the inguinal region; and iodoTMT-131, lymph nodes extending into the scrotum.

**Fig 9:**
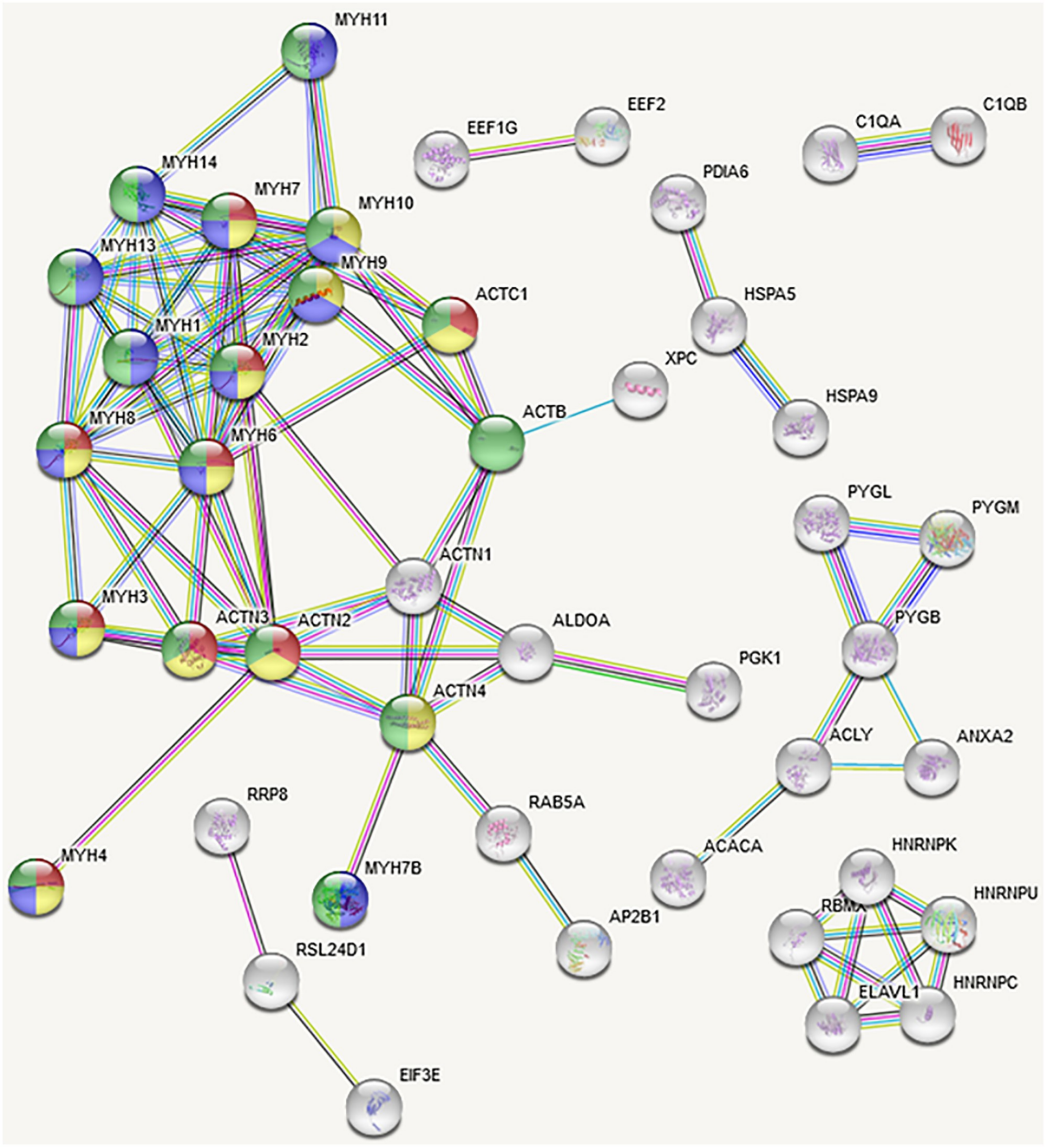
Protein-protein interaction (PPI) networks. Cluster analysis of the proteins in the primary tumors and all metastatic sites had a PPI enrichment p-value of <1.0e-16 indicating the shared SNO-proteins are biologically connected as a group. The reliability of the putative interactions was increased by setting the confidence score at the highest level of 0.900 with a medium (5%) setting for FDR stringency. For secondary analysis, only databased sourced interactions were selected for the network, and all disconnected nodes in the network were eliminated. GO term affiliated with this analysis are as follows. GO:0030049 (orange) muscle filament sliding (1.29e-09), GO:0007018 (blue) microtubule-band movement (5.15e-08); GO:00030048 (yellow) actin filament-based movement (7.48e-10); GO:0006928 (green) movement of cell or subcellular component (7.73e-05).

As shown in S4 File, 331 proteins were identified in primary CDX tumors and distant metastatic sites. Of that total, 60% were identified in all distant metastatic sites with different ratios of expression compared to primary CDX tumors. In contrast, 40% of the proteins from primary CDX tumors were found in some but not all sites. Protein-protein interaction analysis amongst the shared SNO-proteins with PANTHER designated cytoskeletal regulation by the Rho GTPase (P00016) the major pathway for continuity between CDX tumors and metastatic tumors/lymph nodes in *Hltf*-deleted TME. The previously identified secondary pathway of inflammation-mediated by chemokine and cytokine signaling (P00031) now includes SNO-myosins and provides continuity between the primary CDX tumor and all distant locations. Functional enrichment assessment with STRING included actin filament-based process, movement, and binding in conjunction with muscle filament sliding and contraction (Fig 9). These findings strongly implicate S-nitrosylation of actins and myosins under physiological conditions in the metastatic dissemination of CDX tumors in an *Hltf*-deleted TME. Additionally, S-nitrosylation of Cys^813^ in UNC45 myosin chaperone B protein was identified in an iNOS-S1008A/A9-specific motif. UNC45B, a co-chaperone for HSP90 required for folding and accumulation of type II myosins [40] has three tetratricopeptide repeats followed by three armadillo (ARM) repeats. ARM 3 at position 751–790 ends prior to the solitary SNO site at Cys^813^. This SNO-protein is present in all distant metastatic sites except the inguinal region. The NF-kB subunit REL is an example of convergence of transcriptomic-S-nitroso-proteomic signaling. REL is transcriptionally activated (log fold increase 1.768, p = 0.01) in primary CDX tumors from an *Hltf*-deleted TME, and the encoded protein is an S-nitrosylation target (Cys^143^) in all metastatic sites. However, expression is increased (14-fold) in tissue from the inguinal region compared with the primary CDX tumor. Vinculin—a general adhesion protein—dramatically distinguished the metastatic peritoneal carcinomatosis (39-fold) and the lymph nodes extending into the scrotum (168-fold) from the other metastatic sites. However, vinculin appears to be a co-immunoprecipitate because we were unable to authenticate an S-nitrosylated cysteine residue.

## Discussion

The CDX model—male HCT-116 cells in male mice—is predicated on the fact that male gender is a risk factor in metastatic CRC [41]. General risk factors include age, family history of CRC or inflammatory bowel disease, alcoholism, smoking, obesity, and diabetes. Regardless of lifestyle-specific risks, at any age, the incidence of CRC is higher in men [42]. Hormones in the form of oral contraception and hormone replacement therapy protect women from CRC [43]. There is gender disparity in the anatomical location for CRC. Men have a higher risk of developing left-sided (distal to splenic flexure) CRC compared to women who have a higher risk of developing right-sided (proximal to splenic flexure) CRC [44]. Epigenetic silencing of HLTF occurs more often in men [18]. Experimental models such as this CDX model are invaluable as a CRC metastasis model due to the small sample size and sample amount of relevant human tissue, and the limited availability of matched primary and metastatic samples from individual patients. Not to mention the paucity of normal control tissue.

RNAseq and transcriptomic analyses have identified LncRNAs [45, 46] that are gaining importance in cancer genomics studies [47]. LncRNAs—nonprotein coding transcripts >200 nucleotides—are evolutionarily conserved and have been implicated in regulatory specificity through interactions with proteins and/or RNA. LNCipedia (v 5.2) lists 10 Lnc-HLTF transcripts of varying lengths. Lnc-HLTF-1, Lnc-HLTF-2 and Lnc-HLTF-3 are cancer-related although no function is currently assigned to them. In contrast, Lnc-HLTF-5 is elevated in thoracic aorta tissue of patients with hypertension, and positively correlated with expanding ascending aortic diameter [48]. This study is the first to report murine Lnc-Hltf-4 in the *Hltf+/+* TME of a CDX tumor model. It remains to be determined if silencing Lnc-Hltf-4 expression in the *Hltf*-deleted TME actively promoted metastasis.

In this study, the proinflammatory mediator S100A8/A9 in combination with species-specific expression of proinflammatory cytokines IL6 (mouse) and IL8 (human) established a pro-metastatic primary tumor niche in the *Hltf*-deleted TME. Changes in CDX gene expression further promoted metastasis as ICAM-1 increases cancer cell invasion/intravasation into the microvasculature [49] and mediates peritoneal carcinomatosis [50] HP promotes colorectal cancer cell motility [51] and SERPINA3 promotes tumor cell migration and invasion [52]. Decreased HGF may be partly responsible for reduced liver metastasis in this model [53]. Combinatorial induction of IL6 and S100A8/A9 in the TME and ICAM-1 and IL8 in the CDX tumor comprise a positive feedback loop that drives inflammation [54]. These findings support the development of nano-therapeutic tumor-specific and TME-targeted anti-inflammatory therapy for CRC [55, 56].

Increased HP known to preserve vascular NO signaling [57] occurred in conjunction with increased iNOS in the *Hltf*-deleted TME. INOS contributed to the metastatic phenotype via S-nitrosylation of cytoskeletal target proteins (actin, myosin). One of the newest components of the TME under investigation is the mechanical microenvironment [58] with an emphasis on actin [59] especially in invadopodia formation [60] where S-nitrosylation likely plays a regulatory role. The metastatic phenotype was promoted via iNOS-S100A8/A9 site-specific S-nitrosylation of UNC45 myosin chaperone B protein that engages in non-muscle myosin (myosin II) assembly [40]. Most intriguing is the enrichment of vinculin in the two sites of peritoneal metastasis [34]—metastatic peritoneal carcinomatosis [61, 62] and testicular/scrotal metastasis [63]—considered rare and life-threatening. Vinculin, a coimmunoprecipitate—an intracellular F-actin-binding protein and a mechanotransducer in cell-cell and cell-matrix adhesions [64, 65]—has been implicated in tumor progression [66].

Functional protein posttranslational modifications include phosphorylation, ubiquitination and S-nitrosylation. Despite the ongoing search for S-nitrosylation motifs [67], iNOS-S100A8/A9 signaling is the only known process of S-nitrosylation that is site-selective [26]. There are more than 100 putative targets of iNOS-S100A8/A9 nitrosylation. Of those targets, the five with experimentally validated consensus motifs are Annexin A5, Ezrin, GAPDH, Moesin and Vimentin [26]. As a result of this study, POTEE, TRIM52 and UN45B can be added to the list of proteins with authentic SNO sites in the conserved I/L-X-C-X_2_-D/E motif indicative of iNOS-S100A8/A9-mediated S-nitrosylation. Moreover, it appears there is iNOS-S100A8/A9 half-site selective S-nitrosylation. Jai et al. [26] authenticated the iNOS-S100A8/A9 consensus site with *in vitro* mutagenesis experiments in which the target cysteine was changed, or knockdown experiments (siRNA) in which individual components of the heteroduplex (S100A8/A9) were eliminated. However, in this *in vivo* situation, in the presence of both S100A8 and S100A9 proteins, half-site S-nitrosylation was authenticated. Global S-nitrosylation has a role in the regulation of gene transcription [68]. Zinc-fingers motifs are labile to NO where the thio-ligands are vulnerable to S-nitrosylation that can result in zinc release and collapse of the ring-motif resulting in loss of function [69]. HLTF is a zinc finger protein with a highly conserved C3HC4 ring motif (aa760-801); however, we found no evidence of human HLTF-SNO in primary tumors from *Hltf*^+/+^TME. Collectively, these findings support ongoing efforts to find selective iNOS inhibitors as chemo-preventive agents against CRC [23].

## Conclusions

In this study, we show for the first time that *Hltf*-deletion from the TME promotes inflammation in the shared TME-primary tumor niche. Induction of iNOS in the TME produced general and iNOS-S100A8/A9 site-specific S-nitrosylation of previously unidentified human tumor proteins. We establish continuity between global S-nitrosylation of proteins in pathways related to cytoskeleton and motility under physiological conditions in the metastatic dissemination of CDX tumors in an *Hltf*-deleted TME. We provide the first evidence of cross-talk between increased gene transcription and S-nitrosylation of the encoded protein. This new role for *Hltf-*deletion in NO-mediated protein S-nitrosylation to promote metastasis extends our understanding of Hltf as a tumor suppressor.

## Materials and methods

### Reagents and kits

Abcam (Cambridge, MA) was the source of the following antibodies: mouse monoclonal (ab22506) to S100A9 + Calprotectin (S100A8/A9 complex), mouse monoclonal (ab18672) to IL8, rabbit polyclonal anti-iNOS (ab15323), rabbit monoclonal anti-ICAM-1 (ab109361), goat anti-rabbit IgG H&L (HRP; ab97051), goat anti-mouse (HRP; ab205719); and mouse on mouse polymer IHC kit (ab 127055). For immunoprecipitation and Western blotting, Abcam was the source of rabbit polyclonal anti-HLTF (ab17984) to human HLTF aa 600–700 that reacts with mouse and human. For immunohistochemistry, Sigma (St Louis, MO) was the source of rabbit polyclonal anti-HLTF (HPA015284) to human HLTF aa 164–300 that reacts with mouse and human. Biotinylated goat-anti-mouse (BA-9200) and goat anti-rabbit (BA-1000) IgG antibodies and the ABC-enzyme complex were purchased from Vector Laboratories, Inc. (Burlingame, CA). Harris Modified Hematoxylin (HHS16) was purchased from MilliporeSigma (St. Louis, MO). XenoLight D-luciferin potassium salt (122799) was purchased from PerkinElmer (Waltham, MA). ThermoScientific (Waltham, MA) was the source of the following Pierce^™^ reagents: S-nitrosylation Western blot kit (90105), HENS buffer (90106), mouse anti-TMTantibody (90075), immobilized anti-TMT resin (90076), TMT elution buffer (90104) and, iodoTMTsixplex^™^ label reagent set (90101). BioRad (Hercules, CA) was the source of 7.5% Mini-PROTEAN TGX precast protein gels (4561024), Clarity Western ECL-substrate (170–5060), Precision Protein StrepTactin-HRP conjugate (1610381) and Kaleidoscope SDS-PAGE Standards (1610324). PerkinElmer was the source of Bioware^®^ Brite Cell Line HCT116 Red-FLuc (BW124318). All protocols are accessible in protocols.io (dx.doi.org/10.17504/protocols.io.bs5xng7n).

### Cell culture

Bioware^®^ Brite Cell Line HCT116 Red-FLuc is a cell line derived from the parental cell line (ATCC, CCL-247) from adult male colorectal carcinoma by stable transduction with red-shifted lentivirus containing firefly luciferase from Luciola Italica (Red-FLuc) under the control of human ubiquitin C promoter. HCT116 Red-FLuc cells were confirmed to be pathogen free by the IMPACT Profile I (PCR) at the University of Missouri Research Animal Diagnostic and Investigative Laboratory. HCT116 Red-FLuc cells have a mutation in codon 13 of the ras proto-oncogene, and express transforming growth factor beta 1 (TGFβ1) and tumor protein 53 (TP53), HCT116 Red-FLuc cells grown in McCoy’s 5a Modified Medium (ATCC, 30–2007) supplemented with 10% fetal bovine serum and puromycin (2μg/mL) have an average doubling time of 16 hours. For each experiment, cell stocks in liquid nitrogen at passage 2 were thawed using T25 flasks, expanded in T150 flasks for 2 days, passaged overnight and harvested at 70–75% confluency. This protocol provided a unified framework for comparative gene expression analysis.

### *Hltf*-deleted and control mice

Global *Hltf*-deleted mice were developed in collaboration with genOway (Lyon, France) as previously described [35], and bred to be fully congenic (N11) on the C57BL/6J genomic background [36]. Global *Hltf*-deleted mice present a neonatal lethal phenotype. However, when the *Hltf*-deletion line was bred (IACUC# 02007) into the recombinase activating gene 2 (Rag2)/common gamma (IL2rg) double knockout background [37], i.e. mice lacking lymphocytes (NK-, T- B-; alymphoid), the perinatal lethal phenotype was eliminated—clearly showing the perinatal lethal phenotype requires an immune component.

Immune-deficient mice were housed with a 12:12 light/dark cycle with access to food and water *ad libitum* and bedding was changed 2–3 times/week. Routine testing of sentinel mice ensured the colony was disease free. All studies and the anticipated mortality were conducted in accord with the NIH Guidelines for the Care and Use of Laboratory Animals, as reviewed and approved by the Animal Care and Use Committee at Texas Tech University Health Sciences Center (NIH Assurance of Compliance A3056-01; USDA Certification 74-R-0050, Customer 1481, S1 Checklist). TTUHSC’s IACUC (# 02009) specifically approved this study.

The orthotopic *HLTF*^*+/+*^HCT116 xenograft model was established as follows: randomly selected six- to eight-week old *Hltf-deleted* (n = 20) and *HLTF*^*+/+*^ (n = 12) male *Rag2*^*-/-*^*IL2rg*^*-/-*^ mice received direct orthotopic cell microinjections (OCMI) of *HLTF*^*+/+*^HCT116 Red-Fluc cells (2x10^6^ cells/10 μl) between the mucosa and the muscularis layers of the cecal wall. Hereafter the mice were designated *Hltf*-deleted and control. All surgery was performed with isoflurane (Isothesia) and the SomnoSuite^®^ Low-Flow anesthesia system (Kent Scientific) with far infrared warming pads during surgery and recovery. Additional efforts to minimize suffering included an IP injection of Buprenorphine (Buprenex, 0.1 mg/kg) prior to surgery to manage incisional pain followed by a second dose 4–8 hours later. The cecum was exteriorized via a small midline laparotomy on the vertical linea alba to eliminate bleeding. Non-invasive bioluminescence imaging (BLI) with an IVIS Spectrum *In Vivo* Imaging System was used to validate the quality and accuracy of the injection, and to track and quantify tumor growth and metastasis. Histopathology at necropsy confirmed placement of the inoculum. Mouse behavior and well-being were monitored daily. Tumor growth/metastasis was monitored weekly with BLI.

### Techniques

#### Postmortem analysis

Necropsy was performed at 35 days post CDX establishment. Mice under continuous isothesia were imaged and killed immediately (< 15 seconds) by cervical dislocation. Primary tumor xenografts and metastatic tumors were quickly removed, rinsed in physiological saline and either flash frozen for biochemical evaluation (RNA-seq, Western blotting, MALDI-TOF/TOF MS, nanoLC-MS/MS) or fixed in formalin and processed for either routine histopathology or immunohistochemistry.

#### Immunohistochemistry

Tissue blocks were serially sectioned (3–4 μm). Two sections were placed on each slide and deparaffinized prior to staining. Beginning with the first slide, sections on every fifth slide were stained with hematoxylin and eosin (H&E) for evaluation by light microscopy. Sections on alternate slides were processed for immunohistochemistry with heat-induced epitope retrieval. Two tissue sections per slide facilitated the use of one section for positive immunostaining, and the serial section for negative (minus primary antibody) control staining. All primary antibodies (1:50) were paired with an appropriate HRP-conjugated secondary antibody (1:200) depending upon the species in which the primary antibody was generated. Nuclei were counterstained (blue) with hematoxylin.

#### Immunoprecipitation and Western blotting

Immunoprecipitation and Western analysis were performed as previously reported [33, 35]. Briefly, whole cell lysates from two T150 flasks of HCT116 Red-FLuc cells (80% confluent) were immunoprecipitated with rabbit polyclonal anti-HLTF (ab17984) to human HLTF aa 600–700 at a concentration of 5 ug/ml. Western blotting was achieved with the same primary antibody (1:5000) followed by HRP-conjugated mouse anti-rabbit (1:5000). Signal was detected by chemiluminescence with the Clarity Western ECL Substrate Kit.

### Tumor transcriptome analysis (RNA-seq)

Primary tumor xenografts (1 per individual mouse x 3 biological replicates for *Hltf*-deleted and control male mice = 6 total samples) were flash frozen and sent to Otogenetics Corp. (Norcross, GA) for RNA-seq assays as previously described [33, 35–37]. Briefly, total RNA was isolated, and evaluated for its integrity and purity with an Agilent Bioanalyzer (Table 2). RNA samples were rRNA-depleted prior to random-primed cDNA preparation/QC, Illumina library preparation/QC; PE100-125 and HiSeq2500 sequencing at a minimum of 40 million reads. Paired-end 106 nucleotide reads were subjected to species-specific mapping against reference genomes for mouse (mm10) and human (hg38). Differential expression (DE) analysis and alternative splicing analysis for each species was done with cufflinks.cuffdiff (2.2.1). Data were imported into iPathwayGuide (Advaita Corporation, Plymouth, MI) and evaluated using the **q-value of 0.05 for statistical significance** and a log-fold change (logFC) of expression with an absolute value ≤ 0.6. Cuffdiff generated q-values are adjusted p-values that consider the false discovery rate (FDR). The q-value is an essential statistic when measuring thousands of gene expression levels from a relatively small sample set because it has a greater ability (power) to identify significant changes in gene expression. All RNA-seq data in this publication are accessible through NCBI’s Gene Expression Omnibus (GEO) Series accession number GSE161961 (https://www.ncbi.nlm.nih.gov/geo/query/acc.cgi?acc=GSE161961).

**Table 2:** Sample quality control and RNA-seq outcome.

Sample ID	OD260/280	RIN^a^	Total Bases	Total Reads
1-*Hltf*-deleted	2.05	7.7	10,814,132,508	102,026,118
2-*Hltf*-deleted	2.07	6.3	16,571,592,892	156,335,782
3-*Hltf*-deleted	2.07	6.6	8,760,272,904	82,644,084
4-Control	2.10	9.3	4,921,234,440	46,426,740
5-Control	2.09	9.1	4,777,078,680	45,066,780
6-Control	2.10	7.8	6,311,866,884	59,545,914

^a^An RNA integrity number (RIN) from an Agilent Bioanalyzer.

With iPathwayGuide, data were analyzed in the context of pathways obtained from the Kyoto Encyclopedia of Genes and Genomes (KEGG) database (Release 90.0+/05-29, May 19), gene ontologies from the Gene Ontology Consortium database (2019-Apr26), miRNAs from the miRBase (Release 22.1, October 2018) and TARGETSCAN (Targetscan version: Mouse:7.2, Human:7.2) databases, network of regulatory relations from BioGRID: Biological General Repository for Interaction Datasets v3.5.171. March 25th, 2019, and diseases from the KEGG database (Release 90.0+/05-29, May 19).

### Tumor S-nitroso-proteome analysis

Step-wise tumor S-nitroso-proteomic analysis leading to iNOS-S100A8/A9 site-specific analysis was performed with iodoTMT-switch labeling [28], affinity enrichment and high-resolution LC-MS/MS analysis by Applied Biomics, Inc (Hayward, CA). Briefly, primary tumor xenografts from *Hltf*-deleted (n = 2 mice) and control (n = 2 mice) male mice were homogenized/sonicated (Polytron) in 4 volumes HENS buffer (100mM HEPES, pH 7.8, 1 mM EDTA, 0.1 mM Neocuproine, 1% SDS). Protein concentrations were determined (OD_280_) with NanoDrop One (ThermoFisher), and adjusted to a final concentration of 2 μg/μl with HENS buffer. Two hundred microgram samples were incubated for 30 minutes at room temperature with 20 mM methyl methanethiosulfonate (MMTS) in dimethylformamide (DMF) to block free cysteine thiols. Proteins were precipitated with 6 volumes pre-chilled (-20°C) acetone for a minimum of 60 minutes to remove MMTS, pelleted by centrifugation (10,000 x g) for 10 minutes at 4°C, and dried for 10 minutes. Following sample resuspension in HENS buffer (100 μl), S-nitrosylated cysteines were selectively reduced with ascorbate (protected from light) and irreversibly labeled with iodoTMTzero reagent for 2 hours at room temperature. Proteins were precipitated with 6 volumes pre-chilled (-20°C) acetone for a minimum of 60 minutes, pelleted by centrifugation (10,000 x g) for 10 minutes at 4°C, and dried for 10 minutes. Samples were resuspended in HENS buffer (100 μl), added to anti-TMT resin and incubated overnight with end-over-end mixing at 4°C. Resin was washed 3X with 1XTBS, 3X with water, and eluted with 4 volumes TMT elution buffer. Eluates were frozen and lyophilized to dryness. In experiment 1, global S-nitrosylation of CDX tumors from TMT-tagged *Hltf*-deleted and control samples was analyzed by NanoLC-MS/MS with a data base search for human genes in SwissProt using MASCOT. **E**xponentially **M**odified **P**rotein **A**bundance **I**ndex (emPAI defined as 10^PAI^-1) values, where PAI (Protein Abundance Index) is the ratio of observed to observable peptides, is approximately proportional to the logarithm of protein concentration and indicates absolute protein abundance.

In experiment 2, *Hltf*-deleted and control TMT-tagged samples were resolved by two-dimensional gel electrophoresis (2DE) with mouse anti-TMT as the primary antibody (1 μg/ml final concentration) and Cy5-labeled donkey anti-mouse IgG (1:2000) as the second antibody. In experiment 3, TMT-labeled proteins were co-labeled with different color fluorescent dyes, i.e. Cy3 for *Hltf-*deleted and Cy2 for control for two-dimensional difference gel electrophoresis (2D-DIGE), which detected as little as 1.0 fmol of protein in each sample. Spots were excised and proteins identified by matrix-assisted laser-desorption ionization time-of-flight (MALDI-TOF/TOF). Database search was for human proteins in SWISSProt using MASCOT.

In experiment 4, unmodified cysteines in proteins (1 mg/ml) from primary tumor xenografts (n = 4 mice) and metastatic tumors (n = 2 mice) from *Hltf*-deleted mice were blocked with MMTS, selectively reduced with ascorbate (protected from light), and individually tagged with iodoTMTsixplex isobaric reagent such that a unique mass reporter (126–131 Da) in the low-mass region of the MS/MS spectrum was generated for samples from six locations. Equal amounts of six different samples (1 mg each) were combined into a single sample for enrichment with anti-TMT resin and NanoLC-MS/MS analysis. Relative expression for each protein fragment, i.e. the average ratio(s) for the protein, together with the number of peptide ratios that contributed (N) and the geometric standard deviation (SDgeo) were calculated.

In all experiments, peptides labeled with iodoTMT were quantified. Off-target (non-cysteine) labeling was <5%, and authentic cysteine nitrosylation as well as authentic iNOS/S100A8/A9 consensus sequences [26] were confirmed by peptide sequence. Gene symbols for differentially expressed proteins were input to the **P**rotein **AN**alysis **TH**rough **E**volutionary **R**elationships (PANTHER) database [70] for functional classification and pathway identification. Protein-protein networks (cluster analysis) constructed with STRING [71] with EMPAI values identified direct (physical) and indirect (functional) interactions.

## Supporting information

S1 Checklist ARRIVE guidelines 2.0 (fillable). (PDF)

S1 Table Met-analysis gene summary identified 30 DE genes in mouse TME. Cuffdiff, part of Cufflinks, uses RPKM values to calculate changes in gene expression. Cuffdiff data were input into iPathwayGuide. Met-analysis calculated long fold change (logFC) and an adjusted p-value (adjpv) for each comparison. The minus sign in the column for logFC shows 12 genes were downregulated and 18 genes were upregulated.
(XLSX)

S2 Table Met-analysis gene summary identified 151 DE genes in CDX tumor cells. Cuffdiff, part of Cufflinks, uses RPKM values to calculate changes in gene expression. Cuffdiff data were input into iPathwayGuide. Met-analysis calculated long fold change (logFC) and an adjusted p-value (adjpv) for each comparison. The minus sign in the column for logFC shows 136 genes were downregulated and 15 genes were upregulated.
(CSV)

S1 File Excel files for control, test (*Hltf*-deleted TME) and summary of the first experiment in tumor S-nitroso-proteome analysis. Control and test files each contain three sections: section 1 is the legend (how to read the data sheets); section 2 is the protein peptide summary with peptide sequence, and section 3 is the list of significant hits (95% confidence). The summary file contains a comparative list of proteins with emPAI values for control or test or both.
(ZIP)

S2 File Data from the second and third experiment in tumor S-nitroso-proteome analysis. Western blot analysis of test and control samples, 2D-DIGE analysis, and spot identification. Spots were excised and proteins identified by matrix-assisted laser-desorption ionization time-of-flight (MALDI-TOF/TOF).
(ZIP)

S3 File Excel files for spot analysis by MALDI-TOF/TOF. Analysis was conducted in two phases in which spots with a unique or strong signal were analyzed first followed by analysis of spots with a very weak signal Two files each have a section on how to read MS report, a protein peptide summary and a protein ID summary. Only high confidence matches are reported.
(ZIP)

S4 File Excel files for proteins shared by primary and metastatic tumors. There is a legend showing how to read the file, a peptide list and a protein list. The protein list contains ratio data show the relationship of protein expression in the secondary metastatic sites to the primary CDX tumor in the *Hltf-*deleted TME.
(ZIP)
